# Habitual coffee consumption and risk of type 2 diabetes, ischemic heart disease, depression and Alzheimer’s disease: a Mendelian randomization study

**DOI:** 10.1038/srep36500

**Published:** 2016-11-15

**Authors:** Man Ki Kwok, Gabriel M. Leung, C. Mary Schooling

**Affiliations:** 1 School of Public Health, Li Ka Shing Faculty of Medicine, The University of Hong Kong, Hong Kong Special Administrative Region, China; 2 City University of New York Graduate School of Public Health and Health Policy, New York, United States

## Abstract

Observationally, coffee is inversely associated with type 2 diabetes mellitus (T2DM), depression and Alzheimer’s disease, but not ischemic heart disease (IHD). Coffee features as possibly protective in the 2015 Dietary Guidelines for Americans. Short-term trials suggest coffee has neutral effect on most glycemic traits, but raises lipids and adiponectin. To clarify we compared T2DM, depression, Alzheimer’s disease, and IHD and its risk factors by genetically predicted coffee consumption using two-sample Mendelian randomization applied to large extensively genotyped case-control and cross-sectional studies. Childhood cognition was used as a negative control outcome. Genetically predicted coffee consumption was not associated with T2DM (odds ratio (OR) 1.02, 95% confidence interval (CI) 0.76 to 1.36), depression (0.89, 95% CI 0.66 to 1.21), Alzheimer’s disease (1.17, 95% CI 0.96 to 1.43), IHD (0.96, 95% CI 0.80 to 1.14), lipids, glycemic traits, adiposity or adiponectin. Coffee was unrelated to childhood cognition. Consistent with observational studies, coffee was unrelated to IHD, and, as expected, childhood cognition. However, contrary to observational findings, coffee may not have beneficial effects on T2DM, depression or Alzheimer’s disease. These findings clarify the role of coffee with relevance to dietary guidelines and suggest interventions to prevent these complex chronic diseases should be sought elsewhere.

Coffee is habitually consumed in Western societies. Adults in the United States and many European countries typically drink 2 to 3 cups of coffee a day^1^,^2^. With economic development coffee consumption is becoming more common in Asia including South Korea, China and India. Coffee is believed by the general public to have no benefits for type 2 diabetes mellitus (T2DM) or cognitive decline, but to increase the risk of cardiovascular disease (CVD)^3^. In contrast, coffee features in the latest 2015 Dietary Guidelines for Americans as something that might be healthy^4^. Observationally, coffee (both regular and decaffeinated) is monotonically associated with lower risk of type 2 diabetes mellitus (T2DM)^5^. Coffee is also associated with lower risk of depression^6^, as substantiated in a large prospective cohort study of older adults in the United States^7^, as well as of Alzheimer’s disease^8^. Coffee consumption is not clearly associated with ischemic heart disease (IHD)^9^, although moderate coffee drinking may be associated with slightly lower risk^10^. However, observational studies are open to biases from residual confounding by incompletely measured factors that may have major influences on lifestyle and health, such as socio-economic position and health status. Meta-analyses of randomized controlled trials (RCTs) suggest short-term coffee consumption raises triglycerides and low-density lipoprotein (LDL) cholesterol^11^. A small RCT found that short-term coffee consumption increased adiponectin^12^, which may relate to lower CVD risk^13^. Several RCTs showed short-term coffee consumption had no effect on fasting glucose, fasting insulin or insulin resistance^12^,^14^,^15^, although one RCT found it slightly increased glycosylated hemoglobin (HbA1c)^16^. The lack of evidence from long-term RCTs means that the effects of coffee on health are unclear, but are particularly important to establish in this window of opportunity before habitual coffee drinking extends to become the global norm.

In this situation, comparing health by genetically predicted coffee consumption, i.e., using Mendelian randomization (MR), may help clarify the causal effect of coffee on health by generating unbiased estimates from observational studies because MR is less prone to confounding and reverse causality^17^. To date, one MR study, using large cohort studies from Denmark, found no association of genetically predicted coffee consumption with T2DM or CVD risk factors including triglycerides, high-density lipoprotein (HDL) cholesterol, non-fasting glucose, waist circumference and body mass index (BMI)^18^. However, the study was underpowered to assess the effect of coffee on CVD risk factors and did not assess the effect on IHD. To clarify the role of coffee in health, we assessed the role of coffee consumption in T2DM, IHD, CVD risk factors (lipids, glycemic traits, adiposity and adiponectin), depression and Alzheimer’s disease using genetic determinants of coffee from genome-wide association studies (GWAS) applied to very large extensively genotyped case-control and cross-sectional studies. We used childhood cognition as a negative control outcome because coffee unlikely affects cognition in childhood, given coffee drinking usually becomes a habit after adolescence^19^.

## Results

### Genetically predicted coffee consumption

**Figure 1:**
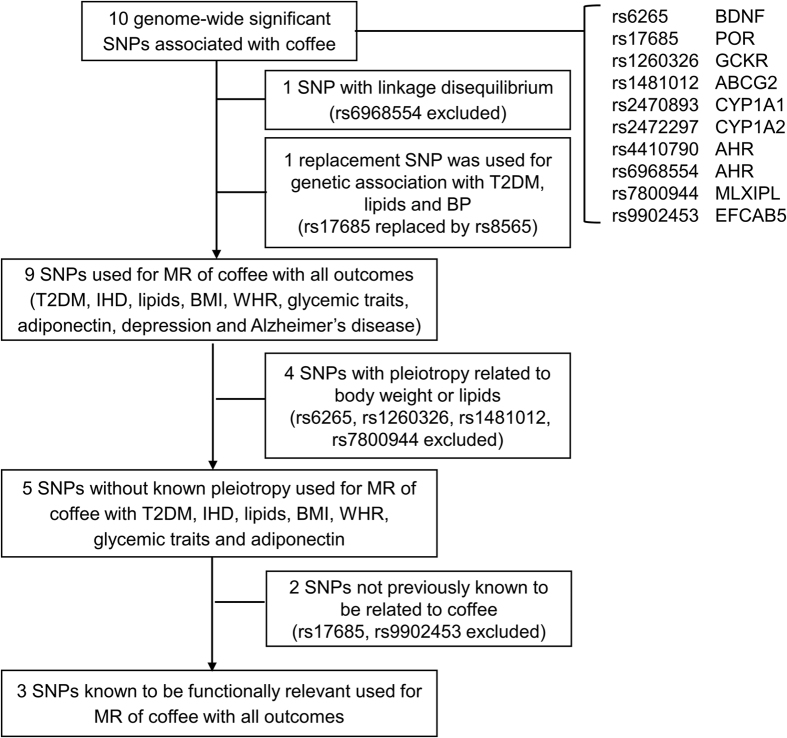
Selection of single nucleotide polymorphisms for Mendelian randomization analysis of the association of coffee consumption with type 2 diabetes mellitus, ischemic heart disease, cardiovascular disease risk factors, depression and Alzheimer’s disease. Abbreviations: HbA1c, glycosylated hemoglobin; HDL-cholesterol, high-density lipoprotein cholesterol; IHD, ischemic heart disease; LDL-cholesterol, low-density lipoprotein cholesterol; MR, Mendelian randomization; SD, standard deviation; SNP, single nucleotide polymorphisms; T2DM, type 2 diabetes mellitus; WHR, waist-hip ratio.

Table 1 shows ten single nucleotide polymorphisms (SNPs) were associated with habitual coffee consumption (number of cups of mainly regular-type coffee per day) at genome-wide significant (log_10_ Bayes Factor > 5.64 which approximates *P* < 5 × 10^−8^) in a GWAS of 129,788 coffee drinkers of mainly European descent (n = 121,824, 94%), mean age 54.0 years^20^. rs6968554 was excluded due to high linkage disequilibrium with rs4410790, giving 9 SNPs. rs17685 was not available for T2DM, lipids, so rs8565 was used instead because it was highly correlated with rs17685 (r^2^ = 0.845), in close proximity (distance within 25 kb of rs17685), had a similar allele frequency (HapMap CEU: rs8565 A (0.29) and rs17685 G (0.71)) and similar genetic association with IHD (Fig. 1). Four SNPs were related to body weight or lipids (rs6265, rs1260326, rs1481012 and rs7800944), so these were excluded for the analyses without known pleiotropy for T2DM, IHD and CVD risk factors. Three non-pleiotropic SNPs, which are known to be functionally relevant to coffee metabolism (rs4410790, rs2472297 and rs2470893)^21^,^22^, were included in the analyses of functionally relevant SNPs. rs2470893 and rs7800944 were not available for childhood cognition, so rs2472297 and rs14415, respectively, were used instead because they are highly correlated with the original SNPs (rs2472297: r^2^ = 0.694; rs14415: r^2^ = 0.816), in close proximity (rs2472297: distance within 10 kb of rs2470893; rs14415: distance within 100 kb of rs7800944) and had a similar allele frequency (HapMap CEU: rs2472297 T (0.25) and rs2470893 T (0.26); rs2286276 T (0.30) and rs7800944 T (0.29)).

Table 2 shows genetically predicted coffee consumption was not clearly associated with T2DM, IHD, depression or Alzheimer’s disease both including and excluding SNPs with known pleiotropy. Most of the estimates were close to the null, particularly after excluding potentially pleiotropic SNPs, although the estimate for Alzheimer’s disease was in a positive direction. Coffee consumption was not clearly associated with most CVD risk factors (lipids, glycemic traits, BMI, WHR and adiponectin) particularly after excluding SNPs with known pleiotropy, although the estimates for LDL-cholesterol, BMI, WHR and adiponectin were in a positive direction. Coffee was unrelated to childhood cognition. An analysis using only the 3 functionally relevant SNPs gave a similar pattern of associations. Not using rs8565 as a replacement for rs17685 gave a very similar pattern of associations (data not shown). The associations remained similar after adjustment for multiple comparison (data not shown).

## Discussion

Consistent with the previous smaller MR study using five SNPs for coffee^18^, we found little evidence of coffee being clearly related to T2DM or major CVD risk factors (HDL-cholesterol, LDL-cholesterol, triglycerides and BMI), although we cannot rule out the possibility of coffee raising LDL-cholesterol, BMI, WHR and adiponectin. Our study adds by replicating these findings in larger samples using more SNPs for coffee and showing coffee was also most likely unassociated with IHD and with glycemic traits, consistent with most^12^,^14^,^15^ but not all^16^ RCTs. This study also adds by showing coffee most likely unrelated to depression and Alzheimer’s disease, although we cannot exclude the possibility that coffee increases the risk of Alzheimer’s disease. Coffee was unrelated to childhood cognition as expected.

This large MR study taking advantage of publicly available ‘big data’ provides more precise estimates with greater statistical power because of the large sample sizes and less susceptibility to weak instrument bias from using 9 SNPs which reduces the possibility of false positives. Nonetheless, limitations exist. First, MR estimates could be confounded by population stratification^23^. We used genetic determinants of coffee from people of predominantly European ancestry (94%) and genetic associations with diseases or its risk factors from people almost exclusively of European ancestry with estimates adjusted for genomic control. In addition, genetic variants predicting coffee are not known to vary geographically within these populations^20^, unlike another beverage, milk, whose genetic determinant, lactase persistence, has a north-south gradient^24^. As such, our MR estimates are unlikely confounded by population stratification. Second, effects of genetic determinants of coffee via pathways other than through coffee intake may generate a bias (by violating the exclusion-restriction assumption)^25^. However, MR estimates with and without pleiotropic SNPs were fairly similar and we placed greater emphasis on the estimates without pleiotropic SNPs. We might have missed some pleiotropic effects because we could only identify known effects and current understanding of the underlying causal pathways. Nonetheless, 3 non-pleiotropic SNPs (rs4410790, rs2472297 and rs2470893) are known to be functionally relevant to coffee metabolism^21^,^22^. An analysis using only these SNPs gave broadly similar results. Third, the genetic variants for coffee were associated with number of cups of coffee per day among coffee drinkers, and the estimates would not relate to the effects of coffee if coffee drinking was uncommon in the samples with the outcomes^26^. However, the populations with the outcomes are from the United States or European countries^27^,^28^,^29^,^30^,^31^ where coffee drinking is typical^1^,^2^. Fourth, we cannot rule out the possibility of a non-linear effect of coffee, although that would require a more complex biological explanation. Fifth, the effect of coffee may vary by sex, given a cohort study found coffee consumption was associated with lower risk of cognitive decline in women but not in men^32^. Whether habitual coffee consumption affects health differently by age, sex or baseline coffee consumption could not be tested because genetic associations with coffee and with the outcomes were obtained from separate samples; however the effects of causal factors are generally consistent, although sex-specific mechanistic pathways are possible. Sixth, we used genetic variants for habitual coffee consumption among coffee drinkers. Whether the findings generalize to ever/never coffee drinkers remains elusive, although extrapolating associations from very infrequent coffee drinkers to never coffee drinkers may be reasonable. Seventh, given coffee drinking usually starts in adulthood, developmental canalization buffering the genetic effects as a compensatory mechanism is unlikely to affect interpretation of the MR estimates. Eighth, participants in the studies used may have taken medication for chronic diseases, although genetic associations with lipids^33^ and glycemic traits were based on participants not taking relevant medication^34^,^35^. However, medication use is unlikely to confound the association of genetic variants with the outcomes, because genetic variants are allocated at conception and precede medication use. Medication use might make the association of genetic variants with coffee consumption less precise. As such, medication use could bias the MR estimate away from the null, hence MR estimates are best interpreted as indicating direction rather than exact effects, particularly for estimates that differ from the null value^36^. Finally, since coffee consumption was not measured in the samples with the outcomes, two-sample MR generates approximate estimates by assuming the genetic associations for coffee are similar in the samples of genetic determinants of coffee and the outcomes^26^. Nonetheless, separate sample MR is more robust to chance findings than single-sample MR because it reduces the possibility of confounding by some cryptic data structure in the single sample^37^.

Unlike previous observational studies^5^, our study, as well as the previous smaller MR study^18^, did not find coffee consumption associated with lower risk of T2DM. Also, unlike some prospective cohort studies^9^,^10^, we found no association of coffee consumption with IHD. Such discrepancies might be partly explained by over-adjustment for potentially harmful mediators, such as BMI or lipids^10^, and the inevitable confounding in observational studies. For CVD risk factors, as in the other MR study^18^, we found little evidence of an association of coffee with HDL-cholesterol or triglycerides. The associations of coffee with LDL-cholesterol and adiponectin are directionally consistent with those found in RCTs^11^,^12^, but do not exclude no association. We also found no association of coffee with HbA1c, fasting glucose, fasting insulin, beta-cell function or insulin resistance, consistent with most^12^,^14^,^15^ but not all^16^ RCTs. In addition, trends in coffee consumption do not coincide with the changing patterns of IHD or T2DM, for example IHD declined^38^ but DM rose^39^ in the United States where coffee consumption was stable in the past decade^40^. Taken together, the overall lack of association of coffee with T2DM, IHD and many CVD risk factors are coherent within this study, and suggest that coffee has likely minor effects, if any, on these conditions.

Our MR study has some consistency with RCTs, although an MR study tests a causal pathway rather than an intervention^41^. Findings from MR give the lifetime effect of coffee and may be more relevant to the health implications of coffee than findings from RCTs evaluating the short-term effect of a coffee intervention^42^. Nonetheless, replication in a larger sample would be valuable. Our findings, using genetic variants for ‘regular’ coffee, i.e., coffee without decaffeination and/or filtration, do not exclude the possibility of coffee raising LDL cholesterol. Coffee has been thought to have cholesterol-raising effects due to the presence of diterpenes (cafestol and kahweol), and such effect is usually removed only when coffee is filtered^43^. Several SNPs functionally relevant to coffee regulate the cytochrome P-450 (CYP) enzyme, which may have implications for CVD risk^44^, but includes a large family of enzymes with different functions. The aryl hydrocarbon receptor (AHR) (rs4410790) regulates CYP1A2 (rs2472297). CYP1A2 is primarily responsible for metabolizing caffeine^21^ and CYP1A1 (rs2470893) metabolizes polycyclic aromatic hydrocarbons, another key ingredient of coffee^22^. CYP1A1/1A2/1B1 knockout mice have lower cholesterol^45^. Whether AHR is related to circulating cholesterol remains elusive; AHR knockout mice have higher hepatic triglycerides in response to high-fat diet^46^. However, SNPs from CYP1A1/2 have not featured in GWAS of CVD or diabetes^27^,^28^,^29^,^47^, consistent with the lack of association with these two conditions.

This study adds by showing no protective association of habitual coffee consumption with depression or Alzheimer’s disease, contrary to meta-analyses of observational studies where coffee is associated with lower risk^6^,^8^. These findings are consistent with null association of coffee with childhood cognition (control outcome). Observed associations of coffee with (particularly subjective measures of) mental health are prone to confounding by socioeconomic position and related attributes (diet and lifestyle), underlying physical health status, and reverse causality. However, the potentially positive association of coffee with Alzheimer’s disease does warrant further investigation. Coffee drinking habits may have changed over time; observationally increasing coffee consumption is associated with higher risk of mild cognitive impairment^48^, while constant moderate coffee consumption is associated with lower risk^48^. Hence, we cannot rule out the possibility that our finding was generated by increased coffee consumption as self-medication for cognitive lapses, although use of genetically predicted coffee consumption should reduce such ‘reverse causality’. Previous observational studies suggest coffee as a modifiable lifestyle factor that may be associated with lower risk of cognitive impairment/decline, although not across all studied cognitive domains^49^,^50^. In addition, cohort studies with more complete follow-up tended to observe weaker negative or positive associations of coffee with dementia^51^. Our MR findings raise a question as to the role of coffee in Alzheimer’s disease, which requires replication, so as to clarify the role of coffee as a potential intervention. Coffee consumption has been associated with smaller volume of the hippocampus and poor memory function^52^. EFCAB5 (rs9902453) is a newly identified SNP for coffee, downstream of SLC6A4, which encodes the serotonin transporter and could reduce circulating serotonin^53^, which might be related to Alzheimer’s disease^54^. Better understanding of whether and how serotonin regulation counteracts neurotoxicity reduction by caffeine induced blockage of adenosine A2 receptor^55^ or other non-caffeine components including chlorogenic acids that have been associated with lower risks of dementia^56^ would help clarify the etiology.

In summary, habitual coffee consumption may not have the beneficial effects on IHD, T2DM, most CVD risk factors, depression and Alzheimer’s disease suggested by observational studies, instead our study raises the possibility that coffee could increase the risk of Alzheimer’s disease and possibly have some unfavourable effects on lipids. This study demonstrates the pitfalls of formulating dietary recommendations based on observational evidence^23^ and emphasizes the importance of genetic validation of potential targets of intervention before making policy or testing interventions^36^.

## Methods

### Genetically predicted coffee consumption

Genetically predicted coffee consumption was based on single nucleotide polymorphisms (SNPs) of genome-wide significant (*P* < 5 × 10^−8^). Highly correlated SNPs (high linkage disequilibrium) (r^2^ > 0.8) were discarded based on larger *P* value with the correlations taken from SNP Annotation and Proxy Search (SNAP) (www.broadinstitute.org/mpg/snap/ldsearchpw.php) using the relevant catalog. SNPs potentially affecting an outcome directly rather than via coffee consumption (pleiotropic effects) were identified from Ensembl (Homo sapiens – phenotype) (http://grch37.ensembl.org/Homo_sapiens/Info/Index). Any SNP for coffee not available for an outcome was replaced with a highly correlated SNP (r^2^ > 0.8).

### Genetically predicted T2DM, IHD, CVD risk factors, depression and Alzheimer’s disease

Genetic associations for T2DM were obtained from the DIAbetes Genetics Replication And Meta-analysis (DIAGRAM), a case (n = 34,840)-control (n = 114,981) study of T2DM mainly in people of European descent (n = 146,171, 98%), mean age 56.9 years, with genomic control and adjustment for study-specific covariates^29^. Data on coronary artery disease/myocardial infarction (MI) have been contributed by CARDIoGRAMplusC4D investigators and have been downloaded from www.CARDIOGRAMPLUSC4D.ORG. CARDIoGRAMplusC4D 1000 Genomes-based GWAS is a case (n = 60,801)-control (n = 123,504) study of IHD and MI in people of European (n = 143,485, 77%), South Asian (n = 25,557, 13%), East Asian (n = 11,323, 6%) and Hispanic or African American descent (~4%), adjusted for age and sex and corrected for genomic control^47^. CARDIoGRAMplusC4D Metabochip is a case (n = 63,746)-control (n = 130,681) study of IHD mainly in people of European descent (n = 176,892, 91%), mean age 57.4 years, adjusted for age and sex and corrected for genomic control^27^. When a SNP was not available in CARDIoGRAMplusC4D, genetic associations were obtained from CARDIoGRAM, a more extensively genotyped subset case (n = 22,233)-control (n = 64,762) study of IHD in people of European descent, mean age 58.1 years, with genetic associations similarly adjusted^28^. Genetic associations for lipids were obtained from the Global Lipids Genetics Consortium (GLGC) which has inverse normal transformed HDL-cholesterol, LDL-cholesterol and triglycerides for 188,577 people of European descent^33^. MAGIC concerns people mainly of European descent without diabetes and has glycosylated hemoglobin (HbA1c) (%) for 46,368 adults^35^, fasting glucose (mmol/L) for 133,010 and log-transformed fasting insulin for 108,557^34^ (or if not available, fasting glucose for 46,186 and fasting insulin for 38,238 based on the 2010 version^57^), homeostatic model assessment (HOMA) β-cell function for 36,466 and HOMA insulin resistance for 37,037^57^. Genetic associations for adiposity were obtained from the Genetic Investigation of Anthropometric Traits (GIANT) which has inverse normal transformed BMI (n = 322,154)^58^ and WHR (n = 210,088) for people of European descent^59^. Genetic associations for adiponectin were obtained from the ADIPOGen Consortium which includes 35,355 people mainly of European descent (n = 29,347, 83%)^60^. Genetic associations for depression were obtained from the Psychiatric GWAS Consortium (PGC), a case (n = 9,240)-control (n = 9,519) study of major depressive disorder in people of European descent, mean age 45.9 years^30^. Genetic associations for Alzheimer’s disease were obtained from the International Genomics of Alzheimer’s Project (IGAP), a case (n = 17,008)-control (n = 37,154) study of Alzheimer’s disease in people of European descent, mean age 71.4 years^31^.

### Genetically predicted childhood cognition (control outcome)

Genetic associations for childhood cognition were obtained from the Social Science Genetic Association Consortium (SSGAC), which has cognition measured by general cognitive ability or intelligence quotient for 17,989 people of European descent^61^.

### Statistical Analysis

Genetic associations with T2DM, IHD, CVD risk factors (lipids, glycemic traits, BMI, WHR, and adiponectin), depression, Alzheimer’s disease and childhood cognition (control outcome) were extracted based on the SNPs predicting habitual coffee consumption. Associations of coffee consumption with these outcomes were obtained using weighted generalized linear regression for correlated SNPs^62^, with a correlation matrix to account for correlation between genetic variants obtained from SNAP using the same catalog as used in the GWAS of the outcome^62^. Given the two IHD case-control studies overlap (57.5% of the cases and 40.1% of controls)^47^, we also combined their results for IHD accounting for this overlap using the Lin and Sullivan approach^63^. Estimates are shown with all genome-wide significant SNPs with potentially pleiotropic effects included and excluded. Estimates are also shown only for non-pleiotropic SNPs known to be functionally relevant to coffee metabolism^21^,^22^. As a sensitivity analysis, given the number of outcomes considered, adjustment was also made for multiple comparisons, using a Bonferroni corrected significance level of 0.002 (0.05/18) to account for testing 18 associations (coffee with four disease outcomes, 13 CVD risk factors and one control outcome).

The statistical analyses were conducted using Stata version 13.1 (StataCorp LP, College Station, TX) and R version 3.2.1 (R Foundation for Statistical Computing, Vienna, Austria).

### Ethics approval

The methods were carried out in accordance with the approved guidelines. People of predominantly European descent were included in the study. Each study has been specifically approved by the Ethical Committees of the original studies and all the participants provided a written informed consent. This analysis of publicly available summary data does not require ethical approval.

## Additional Information

**How to cite this article**: Kwok, M. K. *et al.* Habitual coffee consumption and risk of type 2 diabetes, ischemic heart disease, depression and Alzheimer’s disease: a Mendelian randomization study. *Sci. Rep.* **6**, 36500; doi: 10.1038/srep36500 (2016).

**Publisher’s note**: Springer Nature remains neutral with regard to jurisdictional claims in published maps and institutional affiliations.

## Figures and Tables

**Table 1:** Single nucleotide polymorphisms (SNPs) associated with habitual coffee consumption (mainly regular-type coffee in cups per day) among European and African American coffee drinkers and considered for Mendelian randomization (MR) analyses given they reach genome-wide significance (log_10_Bayes factor > 5.64 which approximates to P < 5 × 10^−8^)^a^ and linkage equilibrium (r^2^ < 0.8).

SNP	Locus	Closest gene	Effect allele	Non-effect allele	Allelic frequency		Habitual coffee consumption			Pleiotropy^b^
					European	African American	beta	SD	*P* value	
rs6265	11p13	BDNF	C	T	0.18	0.07	0.04	0.01	2.69 × 10^−6^	Body mass index, body weight, smoking
rs17685*	7q11.23	POR	A	G	0.30	0.19	0.07	0.01	4.26 × 10^−11^	Nil
rs1260326	2p24	GCKR	C	T	0.36	0.17	0.04	0.01	7.14 × 10^−8^	Cholesterol, triglycerides, kidney diseases, C-reactive protein, glucose tolerance test, platelet count, blood proteins
rs1481012	4q22	ABCG2	A	G	0.89	0.95	0.06	0.01	8.93 × 10^−8^	Gout, response to statin therapy
rs2470893**	15q24	CYP1A1	T	C	0.32	0.06	0.12	0.01	2.72 × 10^−19^	Nil
rs2472297	15q24	CYP1A2	T	C	0.26	0.06	0.14	0.01	2.47 × 10^−24^	Nil
rs4410790	7p21	AHR	C	T	0.35	0.52	0.10	0.01	3.08 × 10^−17^	Nil
rs6968554***	7p21	AHR	G	A	0.39	0.33	0.10	0.01	5.23 × 10^−17^	Nil
rs7800944**	7q11.23	MLXIPL	C	T	0.72	0.67	0.05	0.01	2.29 × 10^−11^	Triglycerides
rs9902453	17q11.2	EFCAB5	G	A	0.53	0.80	0.03	0.01	2.44 × 10^−8^	Nil

Abbreviations: MR, Mendelian randomization; SNP, single nucleotide polymorphisms.

^*^rs17685 was not available for type 2 diabetes mellitus and lipids, so rs8565 was used instead because it was highly correlated with rs17685 (r^2^ = 0.845), in close proximity (distance within 25 kb of rs17685), had similar allele frequency (HapMap CEU: rs8565 A (0.29) and rs17685 A (0.30)) and similar genetic association for ischemic heart disease.

^**^rs2470893 and rs7800944 were not available for cognition. For rs2470893, rs2472297 was used instead because it was highly correlated with rs2470893 (r^2^ = 0.694), in close proximity (distance within 10 kb of rs2470893) and had similar allele frequency (HapMap CEU: rs2472297 T (0.25) and rs2470893 T (0.26)). For rs7800944, rs14415 was used instead because it was highly correlated with rs7800944 (r2 = 0.816), in close proximity (distance within 100 kb of rs7800944) and had similar allele frequency (HapMap CEU: rs2286276 T (0.30) and rs7800944 T (0.29)).

^***^rs6968554 reaches genome-wide significance but was excluded from the analyses because of linkage disequilibrium with rs4410790 and larger P value.

^a^Reference: The Coffee and Caffeine Genetics Consortium, Cornelis MC, Byrne EM, Esko T, Nalls MA, Ganna A *et al*. Genome-wide meta-analysis identifies six novel loci associated with habitual coffee consumption. Mol Psychiatry. 2015; 20:647–56.

^b^Pleiotropy was identified using Ensembl (Homo sapiens – phenotype) (http://grch37.ensembl.org/Homo_sapiens/Info/Index).

**Table 2:** Association of genetically predicted habitual coffee consumption with type 2 diabetes mellitus, ischemic heart disease, cardiovascular disease risk factors, depression and Alzheimer’s disease obtained from Mendelian randomization analyses using weighted generalized linear regression.

Outcomes	Consortium	All 9 SNPs				3 functionally relevant SNPs^a^	
		All genome-wide significant SNPs^a^		5 SNPs without known pleiotropy related to body weight or lipids^a^		Odds ratio	95% CIs
		Odds ratio	95% CIs	Odds ratio	95% CIs		
**Diseases**							
Type 2 diabetes mellitus*	DIAGRAM	**1.20**	**1.00, 1.42**	1.03	0.81, 1.31	1.05	0.70, 1.55
Ischemic heart disease	CARDIoGRAMplusC4D 1000 Genomes-based GWAS	1.06	0.94, 1.20	1.07	0.91, 1.26	1.12	0.84, 1.49
	CARDIoGRAMplusC4D Metabochip/CARDIoGRAM	0.96	0.84, 1.10	0.96	0.80, 1.14	0.97	0.73, 1.30
	Pooled	1.02	0.93, 1.12	1.02	0.92, 1.13	1.05	0.94, 1.17
Depression	PGC	0.89	0.66, 1.21	NA		0.95	0.47, 1.91
Alzheimer’s disease	IGAP	1.17	0.96, 1.43	NA		1.29	0.82, 2.03
**Cardiovascular disease risk factors**	Consortium	Mean difference	95% CIs	Mean difference	95% CIs	Mean difference	95% CIs
HDL-cholesterol (SD)*	GLGC	0.04	−0.01, 0.09	−0.02	−0.09, 0.05	−0.02	−0.13, 0.10
LDL-cholesterol (SD)*	GLGC	0.01	−0.04, 0.06	0.06	−0.01, 0.14	0.05	−0.07, 0.17
Triglycerides (SD)*	GLGC	**−0.26**	**−0.31, −0.21**	0.02	−0.05, 0.08	−0.004	−0.11, 0.10
Body mass index (SD)	GIANT	**0.12**	**0.08, 0.17**	0.05	−0.005, 0.11	0.05	−0.04, 0.15
Waist-hip ratio (SD)	GIANT	**0.05**	**0.004, 0.10**	0.06	−0.01, 0.12	0.06	−0.05, 0.16
HbA1c (%)	MAGIC	0.03	−0.02, 0.08	0.01	−0.05, 0.08	0.02	−0.09, 0.13
Fasting glucose (mmol/L)	MAGIC	**0.04**	0.01, 0.08	−0.01	−0.06, 0.03	−0.02	−0.09, 0.05
Fasting insulin (log-transformed)	MAGIC	0.02	−0.02, 0.06	−0.03	−0.09, 0.02	−0.03	−0.11, 0.05
β-cell function (log-transformed)	MAGIC	0.03	−0.02, 0.07	0.03	−0.04, 0.09	0.04	−0.06, 0.15
Insulin resistance (log-transformed)	MAGIC	**0.06**	**0.0005, 0.12**	0.03	−0.05, 0.10	0.04	−0.09, 0.17
Adiponectin (log-transformed)	ADIPOGen	0.04	−0.02, 0.10	0.04	−0.04, 0.12	0.03	−0.11, 0.17
**Control outcome**	Consortium	Mean difference	95% CIs	Mean difference	95% CIs	Mean difference	95% CIs
Childhood cognition**	SSGAC	0.07	−0.09, 0.23	NA		0.10	−0.26, 0.45

Bold indicates statistical significance (*P* < 0.05).

Abbreviations: CI, confidence interval; HbA1c, glycosylated hemoglobin; HDL-cholesterol, high-density lipoprotein cholesterol; LDL-cholesterol, low-density lipoprotein cholesterol; NA, not applicable; SD, standard deviation; SNP, single nucleotide polymorphisms.

^*^rs17685 was not available for type 2 diabetes mellitus and lipids, so rs8565 was used instead because it was highly correlated with rs17685 (r^2^ = 0.845), in close proximity (distance within 25 kb of rs17685), had similar allele frequency (HapMap CEU: rs8565 A (0.29) and rs17685 A (0.30)) and similar genetic association for ischemic heart disease.

^**^rs2470893 and rs7800944 were not available for cognition. For rs2470893, rs2472297 was used instead because it was highly correlated with rs2470893 (r^2^ = 0.694), in close proximity (distance within 10 kb of rs2470893) and had similar allele frequency (HapMap CEU: rs2472297 T (0.25) and rs2470893 T (0.26)). For rs7800944, rs14415 was used instead because it was highly correlated with rs7800944 (r2 = 0.816), in close proximity (distance within 100 kb of rs7800944) and had similar allele frequency (HapMap CEU: rs2286276 T (0.30) and rs7800944 T (0.29)).

^a^All SNPs included for analyses were rs6265, rs17685, rs1260326, rs1481012, rs2470893, rs2472297, rs4410790, rs7800944 and rs9902453; SNPs without known pleiotropy included for analyses were rs17685, rs2470893, rs2472297, rs4410790 and rs9902453; and functionally relevant SNPs included for analyses.
